# Dilemma in Timing of Delivery in a Patient with an Acute Myocardial Infarction

**DOI:** 10.1155/2015/635315

**Published:** 2015-01-08

**Authors:** Laura M. Héman, Ingrid E. C. Devies, Frans J. M. E. Roumen

**Affiliations:** Department of Obstetrics and Gynecology, Atrium Medical Center Parkstad, Henri Dunantstraat 5, 6419 PC Heerlen, Netherlands

## Abstract

*Introduction*. Acute myocardial infarction (AMI) in a pregnant woman is rare. When occurring, AMI is a major cause of maternal and neonatal death. By presenting the following case we describe the dilemma concerning the timing of delivery. *Case*. A 36-year-old, multiparous women, at 35 6/7 weeks of gestation, suffered from an AMI due to an acute blockage of the left anterior descending artery (LAD). This was treated by angiographic thrombosuction and biodegradable stent placement. Within 5 hours after this procedure, a cesarean section (CS) was performed because of a nonreassuring fetal condition. A healthy son with an Apgar score of 9/10 was born. The patient's postoperative course was complicated by a big wound hematoma, a hemoglobin drop, and heart failure. *Discussion*. In case of AMI during pregnancy, the cardiological management has absolute priority. The obstetrical management is not outlined. In a nonreassuring fetal condition, delivery is indicated after stabilization of the mother. However, delivery after recent AMI and angiography will bring new risks of cardiologic stress and bleeding complications. The limited literature available tends to an expectant obstetrical management, but this case emphasizes the difficulty of waiting in suspected fetal distress.

## 1. Introduction

Acute myocardial infarction (AMI) occurs in 2,8–6,2 per 100.000 deliveries [1, 2]. Pregnancy itself increases the risk of AMI 3-4-fold [1]. Hypertension, thrombophilia, diabetes mellitus, smoking, transfusion, postpartum infection, and age of 30 and older ages are significant risk factors for pregnancy related AMI [1].

In the western world, cardiac disease causes 11% of all maternal deaths [3]. Most maternal deaths occur at the time of the infarction (usually resulting in an undelivered child) or within two weeks of infarction (usually in association with labor and delivery) [4]. Fetal mortality is related to maternal death and maternal survival is usually accompanied by normal fetal outcome [4].

In the acute phase, the first concern is to stabilize and treat the mother. The appropriate coronary intervention should be selected. The treatment options, pharmacological, percutaneous coronary intervention (PCI) and/or surgery as well as the anticoagulants used, will influence the decisions on timing and mode of delivery.

In a nonreassuring fetal condition, delivery is indicated after stabilization of the mother. But when can attention be shifted to the fetus, since a delivery will bring new risks of cardiologic stress and anticoagulants-induced bleeding complications for the mother?

By presenting this case, we describe the dilemma we encountered concerning the timing of the delivery and the choices we have made.

## 2. Case

A 36-year-old woman, gravida 4 para 2, amenorrhea of 35 weeks and 6 days, with a history of two uncomplicated deliveries and no thromboembolic events, was presented at the labor ward. She complained of backache (between the scapulas) starting three hours earlier and a pricking and later pressing pain on the chest with paresthetic radiation to the left arm. There was a slight shortness of breath. She felt unwell and vomited once. Her complaints were not recognized as signs of an AMI by the emergency services (by telephone) and the general practitioner who visited her earlier that day. She smoked 15 cigarettes a day. Her father had a cerebral vascular event at the age of 54 and had a hemiparesis. Her grandfather on paternal side died of a heart attack at the age of 74.

On examination, she was apprehensive but not clammy, pale, or dyspnoeic. Blood pressure (BP) was 117/84 mmHg, pulse 75 beats per minute (bpm), temperature 36,2°C, and respiration 20 breaths per minute. On auscultation, there were clear lungs and normal heart sounds. Her legs were not painful and not swollen.

Differentially, we thought of a lung embolism because of a history of a painful left leg, gastric acids, or an ulcer because of preexisting gastric pain and an AMI.

An electrocardiogram (ECG) showed ST elevation in the anteroseptal leads. Laboratory results were Hb 8,5 mmol/L (7,5–10,0), CK 170 U/L (0–160), CK/MB 16,8 ug/L (0,0–2,9), and troponin-T 0,21 ug/L (0,00-0,01). A loading dose of aspirin 500 mg and ticagrelor 80 mg (platelet inhibitor) was given. Urgent angiography via the femoral artery was performed in left lateral tilt within one hour after presentation. It showed a complete obstruction of the left anterior descending artery (LAD). During the PCI, thrombosuction was performed and a biodegradable stent (absorbable bioresorbable vascular scaffold (BVS)) was inserted under 7000 IU heparin and 33 mL tirofiban (glycoprotein IIb/IIIa inhibitor). During the stent placement, the patient developed hypotension which was treated with 500 mL of a plasma expander (Gelofusine). A secondary spasm of the LAD was treated with nitroglycerine intracoronary after which the LAD opened again.

Fetal protection of radiation exposure during angiography was established by means of a lead cover on the patient's back. The total radiation dose was 0,15 gray. Cardiotocography (CTG) registration was done continuously from admission. Maternal hypotension during stent placement (BP 80/50) resulted in two deep decelerations with a fetal heart rate of 50 bpm which continued for 3 and 2 minutes, respectively. Then, the fetal heart rate returned to the baseline of 130 bpm. During the recovery of the mother at the coronary care unit (CCU), the fetal heart rate showed several decelerations of 2 minutes with variable recovery to a baseline of 140 bpm. Relative hypotension of the mother (100/70) was treated with administration of in total 4 × 500 mL plasma expander. Four hours after PCI, despite lateral tilt and a raised BP to 125/85, fetal distress was suspected: the CTG showed a 5-minute deceleration of the fetal heart rate to 50 bpm with a slow recuperation to the baseline. After extensive consultation with the cardiologist, the decision to perform a cesarean section (CS) was made. Two units of platelets were ordered, to be available in case of bleeding problems. The anesthesiologist decided to give general anesthesia duo to spinal bleeding risks. During an uncomplicated procedure, a boy of 2660 gram was born with an Apgar score of 9/10. (The arterial pH was 7,32 and base excess −3,0.) A total of 10 IU of oxytocin was given: 5 IU i.v. stat and 5 IU in sodium chloride to run in four hours. Total blood loss during the CS was estimated at 400 mL, so there was no indication to antagonize the heparin or to give platelets.

The first night postpartum, the patient showed signs of heart failure: dyspnea, hypotension, and pulmonary edema. She improved on furosemide. During admission, she received in total four packed cells and two units of fresh frozen plasma. The hemoglobin (Hb) ranged between 4,9 and 6,8 mmol/L. There was no uterine atony and we never suspected an acute intra-abdominal bleeding. She developed a large wound hematoma at the incision site, probably due to reduced coagulation. Cardiac medication included B-blockage, an ACE-inhibitor, platelet inhibitor, aspirin, spironolactone, furosemide, and a statin. At day six, mother and child were discharged in relatively good health from the hospital.

## 3. Discussion

### 3.1. General Considerations in Acute Management of Pregnant Women with AMI

Symptoms of AMI can be masked or misinterpreted in pregnancy. ECG is a safe, noninvasive diagnostic test to detect cardiac problems. Cardiac markers can help with making the diagnosis: troponin-I is a sensitive marker for MI in pregnancy, where CK-MB serum concentrations can be elevated in pregnancy [5]. Angiographic intervention of acute coronary syndrome in pregnancy is advised above thrombolysis and surgery [6, 7]. Coronary dissection, which is more common in pregnancy, can be found this way [6]. Balloon angioplasty or stent placement is advised in atherosclerotic disease.

When comparing the bare metal stent (BMS) and a drug eluting stent (DES), the period of antiplatelet therapy is to be considered. A DES requires prolonged antiplatelet therapy, while with BMS antiplatelet therapy is necessary for a shorter period of time. Therefore, if delivery can be delayed for more than 4 weeks, BMS is preferred [6, 8]. A biodegradable stent (BVS), which was given in this case, is preferable in young people but does need prolonged antiplatelet therapy [9]. There is no available literature about the use of a BVS in pregnancy.

All anticoagulant and antiplatelet agents increase the risk of bleeding. When heparin is used, a discontinuation of 6 hours is desirable before delivery [6]. Protamine sulphate might be required to antagonize the effect before delivery, operation, and spinal/epidural anesthesia [8]. When using antiplatelet therapy, that is, clopidogrel, ticagrelor, or glycoprotein IIa/IIIb (i.e., Tirofiban), epidural/spinal bleeding complications should be taken into account. Glycoprotein IIa/IIIb might have a potential risk of fetal intracranial hemorrhage during delivery. Roth and Elkayam state that until more research has been done, a CS should be considered when using glycoprotein IIa/IIIb [6]. If clopidogrel is given, it is recommended to stop 5–7 days before CS [6, 10]. Whether it is useful to give prophylactic platelets before delivery is unclear [11].

Vaginal delivery is preferable in maternal cardiac disease [5]. Due to the risk of recurrent myocardial ischemia or even acute heart failure, the delivery should, if possible, be postponed for 2-3 weeks. If this is not possible, a CS could be an option [12]. The advantages of CS are better control of the time of delivery and the avoidance of stressful labor. Disadvantages are the added anesthetical and surgical risks, that is, blood loss, respiratory complications, pain, and infection [6]. During labor, one should be reserved with uterotonic drugs. Ergometrine is contraindicated in hypertension and ischemic heart disease [5]. Oxytocin also proved to have negative cardiovascular effects [13].

### 3.2. The Dilemma

The greatest challenge in this case was to decide on the timing of delivery. In a nonreassuring fetal condition, delivery is indicated after stabilization of the mother. But when is the mother stable enough to endure a CS?

Maternal mortality in patients with AMI is twice as high as that in the peripartum period compared to the ante- or postpartum period. [2, 5, 6]. This favors an expectant policy concerning the delivery. Increased maternal mortality and morbidity peripartum after recent AMI and angiography may be caused by (i) direct cardiac ischemic stress, (ii) bleeding problems due to anticoagulant and antiplatelet treatment, and (iii) the big hemodynamic change which occurs in delivery [4].

As mentioned in the introduction, the first concern is to stabilize and treat the mother. The usefulness of continuous CTG registration is therefore doubtful. Moreover, a nonreassuring fetal condition is probably caused by instability of the mother in the first place, so this should be treated first. In this case, we considered the hypotension to be the cause of the CTG decelerations. However, BP improvement did not give the CTG improvement we expected. Differentially, the deceleration in the fetal heart rate could also be caused by reduced fetomaternal circulation due to maternal heart failure and/or cardiogenic shock. It is very difficult to predict the course of the maternal condition and the chances of acute complications like ventricular fibrillation, heart failure, and cardiac arrest after recent AMI and angiography. If her condition deteriorates, she might even be better off without pregnancy [14]. Another argument pro delivery (by SC) is that it will objectify the fetal condition.

In this case, after deliberation with the cardiologist and anesthesiologist, we did decide to perform the CS due to a nonreassuring fetal condition. The patient, however, developed a large wound hematoma, an Hb drop, and heart failure in the first night postpartum. In retrospect, with respect to the maternal hypotension, we initially could have given inotropic treatment to increase the blood pressure, especially since fluid therapy in a patient with anterior wall involvement can cause heart failure.

It turned out that there was no fetal asphyxia, considering the Apgar score and the pH. This may argue for a more expectant policy after angiography, especially since heparin administration was less than 6 hours. Nevertheless, with all the things considered, we regard the outcome to be successful, since mother and child were able to go home in relative good health within one week.

AMI in pregnancy is a rare and severe condition in which the cardiological management has priority. The obstetrical management is not outlined. The limited literature available tends to an expectant obstetrical management, but this case emphasizes the difficulty of waiting in suspected fetal distress.
